# EGFR tyrosine kinase inhibitors versus chemotherapy as first-line therapy for non-small cell lung cancer patients with the L858R point mutation

**DOI:** 10.1038/srep36371

**Published:** 2016-11-04

**Authors:** Jianlin Xu, Haitang Yang, Bo Jin, Yuqing Lou, Yanwei Zhang, Xueyan Zhang, Hua Zhong, Huiming Wang, Dan Wu, Baohui Han

**Affiliations:** 1 Department of Pulmonary, Shanghai Chest Hospital, Shanghai Jiaotong University, Shanghai 230032, China; 2 Department of Pathology, Shanghai Chest Hospital, Shanghai Jiaotong University, Shanghai 230032, China; 3 Central laboratory, Shanghai Chest Hospital, Shanghai Jiaotong University, Shanghai 230032, China

## Abstract

The efficacy of EGFR tyrosine kinase inhibitors (TKIs) varies among different EGFR mutations. Here, we directly compared the efficacy of first-line TKIs to chemotherapy for non-small cell lung cancer (NSCLC) patients with the L858R mutation. The progression-free survival (PFS) for patients receiving TKIs as first-line therapy was longer than those who received chemotherapy (hazard ratio [HR]: 0.44, P < 0.001). Subgroup analyses showed that first-line TKI therapy resulted in longer PFS among non-smokers (HR: 0.41, P < 0.001), male (HR: 0.49, P = 0.002), female (HR: 0.39, P < 0.001), and patients with adenocarcinoma histology (HR: 0.41, P < 0.001). However, among patients with non-adenocarcinoma histology (HR: 1.11, P = 0.824) and those who used to smoke (HR: 0.55, P = 0.093), first-line TKI therapy failed to demonstrate statistically longer PFS compared to chemotherapy. Our results demonstrated that for patients with L858R mutation, first-line TKI therapy provided better survival benefits. However, among non-adenocarcinoma patients and those who used to smoke, the PFS in cohorts receiving first-line chemotherapy or TKI were not significantly different. The results of the current study will be helpful for decision-making in the treatment of patients with L858R mutation.

Lung cancer is the most frequently diagnosed cancer among men worldwide, and is also the leading cause of cancer-related deaths among women in China^1^,^2^. Platinum-based chemotherapy has been found to provide a survival benefit for patients with advanced lung cancer; however, most patients do not survive longer than 1 year^3^. In the last decade, the discovery of EGFR mutations and subsequent therapies targeting this receptor have changed the treatment patterns and outcomes of non-small cell lung cancer (NSCLC)^4^,^5^. The two most common EGFR mutations are an exon 19 deletion and L858R point mutation, which account for 80–90% of all EGFR mutations^6^. Those two mutations are generally considered sensitive mutations that exhibit a favorable response to tyrosine kinase inhibitors (TKIs)^7^,^8^,^9^,^10^,^11^,^12^. Several studies have reported that advanced NSCLC patients with the L858R mutation had a shorter overall survival (OS) and/or progression-free survival (PFS) following EGFR TKI therapy compared to those with EGFR exon 19 deletion^13^,^14^,^15^. In addition, the LUX-Lung 3 and LUX-Lung 6 trials showed a benefit in OS for patients with exon 19 deletion with the use of afatinib, but no benefit in OS for patients with the L858R mutation^16^. Therefore, we summarized the clinical data of patients who harbored the L858R mutation to directly compare the efficacy of first-line TKIs and chemotherapy for NSCLC patients with the L858R mutation.

## Results

### Patient characteristics

A total of 245 NSCLC patients harboring the L858R mutation with treatment and survival details were included in this analysis, of which 118 patients received EGFR TKIs as first-line therapy, whereas 127 patients received chemotherapy as first-line therapy. Demographic data of all of the patients are shown in Table 1.

### Efficacy

**Figure 1:**
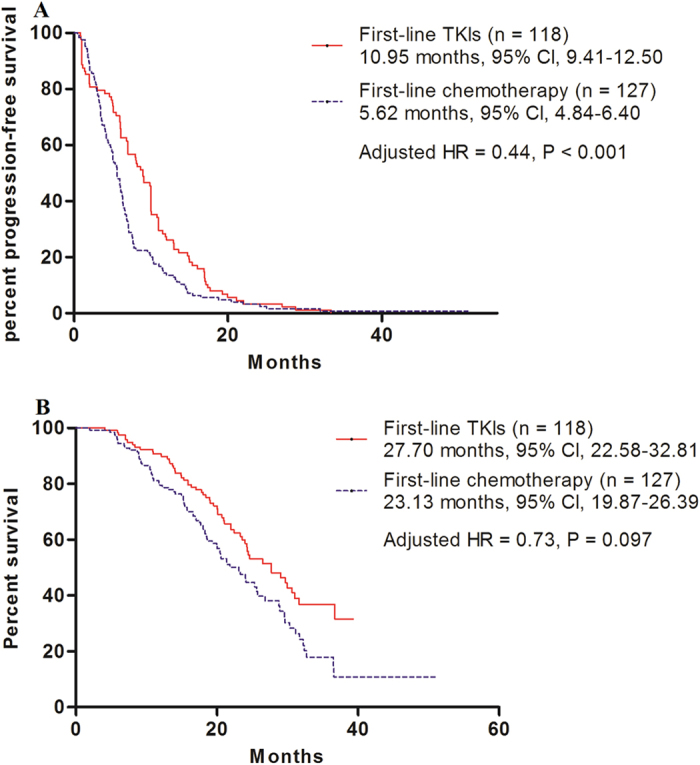
Comparison of progression-free survival (PFS) and overall survival (OS). (**A**) Kaplan–Meier survival curves for PFS analysis between first-line TKI therapy and chemotherapy. (**B**) Kaplan–Meier survival curves for OS analysis between first-line TKI therapy and chemotherapy. TKI, tyrosine kinase inhibitor.

**Figure 2:**
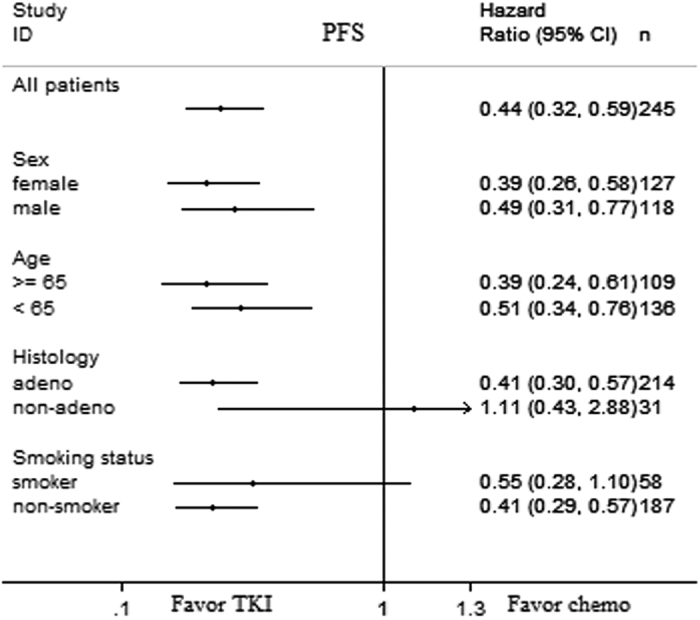
Forest plot of progression-free survival (PFS) by clinical characteristics. First-line TKI therapy versus first-line chemotherapy among patients with different clinical characteristics. TKI, tyrosine kinase inhibitor.

The PFS for patients who received chemotherapy or TKIs as first-line therapy were 5.62 months (95% CI: 4.84–6.40) and 10.95 months (95% CI: 9.41–12.50), respectively (adjusted hazard ratio [HR] = 0.44, 95% CI: 0.32–0.59, P < 0.001) (Fig. 1A). Subgroup analyses showed that first-line TKI therapy led to a longer PFS among non-smokers (adjusted HR = 0.41, 95% CI: 0.29–0.57, P < 0.001), male (HR = 0.49, 95% CI: 0.31–077, P = 0.002), female (HR = 0.39, 95% CI: 0.26–0.58, P < 0.001), and patients with adenocarcinoma histology (HR = 0.41, 95% CI: 0.30–0.57, P < 0.001). However, among patients with non-adenocarcinoma histology and those with a smoking history, first-line TKI therapy failed to demonstrate a statistically longer PFS compared to first-line chemotherapy. The adjusted HRs were 1.11 (95% CI: 0.43–2.88) and 0.55 (95% CI: 0.28–1.10), respectively (Fig. 1B). The OS for patients receiving chemotherapy or TKIs as first-line therapy was 23.13 months (95% CI: 19.87–26.39) and 27.70 months (95% CI: 22.58–32.81), respectively (adjusted HR = 0.73, 95% CI: 0.54–1.06, P = 0.097, Fig. 2).

## Discussion

The efficacy of EGFR TKIs varies among different EGFR mutations^17^. The association of first-line EGFR TKI therapy for advanced NSCLC patients with a specific EGFR mutation genotype remains unclear. In this study, we compared the efficacy of EGFR TKIs and chemotherapy as first-line therapy in patients with the L858R point mutation. The results demonstrated that EGFR TKIs led to a longer PFS compared to chemotherapy among these patients. Previously, a meta-analysis assessed the effects of EGFR TKI for the treatment of patients with the L858R point mutation, and the results were similar to those found in the present study^18^; however, the study did not make subgroup analyses of this population. In the current study, subgroup analyses showed that among smoker and non-adenocarcinoma patients with the L858R point mutation, the PFS of first-line chemotherapy and TKI therapy cohorts were not significantly different.

According to a previous study, smoking status is an independent predictive factor of EGFR TKI treatment outcome in NSCLC patients with sensitive EGFR mutations^19^. Patients who never smoked have a better survival outcome after EGFR TKI therapy compared to those with a smoking history^20^. It has been reported that lung cancer in smokers has multiple genetic alterations that are associated with smoking, such as the activation of AKT and ERK signaling pathways^21^, and those alterations mediate the resistance to EGFR TKIs^22^. In the present study, among the L858R-positive patients with a smoking history, first-line EGFR TKI failed to result in a statistically longer PFS compared to first-line chemotherapy. EGFR mutations can also be detected in nonadenocarcinoma. Specifically, several previous reports have shown that EGFR mutations can be detected in 35.1–44.0%, 3.9–10.0%, and 11.5–14.3% of patients with adenosquamous carcinoma, squamous cell carcinoma and large cell carcinoma, respectively^23^,^24^,^25^. The results of our prospective multicenter study (IGNITE study) demonstrated that in an Asian population with lung squamous cell carcinoma, the EGFR mutation frequency was 10%^26^. However, in this population, the efficacy of EGFR TKIs seemed to be inferior than that in patients with adenocarcinoma histology. A pooled analysis identified EGFR mutation-positive patients in all clinical reports that contained advanced non-adenocarcinoma NSCLC patients harboring EGFR mutations who were treated with gefitinib^27^, and the PFS of those 19 EGFR mutated non-adenocarcinoma patients was 3.0 months which was less than the PFS of adenocarcinoma patients reported in previous reports^7^,^8^,^9^,^10^,^11^,^12^. In the current study, for L858R-positive patients with non-adenocarcinoma histology, first-line TKI therapy failed to result in a longer PFS compared to chemotherapy.

Results from the LUX-Lung 3 and LUX-Lung 6 trials showed a benefit in OS for patients with EGFR del19-positive lung adenocarcinoma with the use of afatinib, but the drug did not result in significant improvements in OS compared to conventional chemotherapy in patients with the L858R mutation^16^. Similarly, in the present study, the OS for L858R-positive patients receiving chemotherapy or TKIs as first-line therapy were also not significantly different. However, although the results were not statistically significant, the cohort receiving first-line TKI seemed to respond better than those receiving first-line chemotherapy, based on Kaplan–Meier curves for OS and PFS. The high crossover rate to second-line or third-line EGFR TKI therapy in the first-line chemotherapy cohort can explain the failure to achieve a statistically longer OS in the first-line EGFR TKI therapy cohort. In the present study, 115 of 127 patients in the first-line chemotherapy cohort received subsequent EGFR TKI therapy. In other words, the PFS benefit of first-line TKIs did not appear to translate into an OS benefit in previous clinical trials. This could be partly explained by the subsequent effect of EGFR TKI therapy on OS. The OPTIMAL study’s final OS results demonstrated that the median OS between the first-line erlotinib arm and the first-line chemotherapy arm was similar^28^. According to the in-depth analysis of the OPTIMAL study, 36.6% of patients with common mutations who received first-line erlotinib did not receive post-study therapy, and 22.2% of patients who received chemotherapy did not receive any post-study treatment, which could partly explain why the first-line erlotinib arm did not show superiority in OS over the first-line chemotherapy arm. In the EURTAC study, the HR of OS for the first-line erlotinib arm versus the first-line chemotherapy arm was 0.92 (95% CI: 0.63–1.35). Considering the effects of post-study treatments on first-line treatment, after using statistical models to control for second line post-study treatment effects, the HR for OS was 0.68 (95% CI: 0.37–1.25)^29^.

The results of this study should be interpreted while keeping several limitations in mind. The major limitation was its retrospective nature. Second, the small sample size in the smoker and non-adenocarcinoma subgroups might have affected the statistical analysis.

In conclusion, for NSCLC patients with L858R mutation, first-line TKI therapy provided better survival benefits. However, among non-adenocarcinoma patients and those who smoked, the PFS of first-line chemotherapy and TKI therapy cohorts were not significantly different. Considering the retrospective nature of this study, a prospective study is needed to confirm the results.

## Patients and Methods

### Patients

This study was approved by Institutional Ethics Committee at Shanghai Chest Hospital. All subjects or their family members provided written informed consent. All procedures were conducted according to the guidelines approved by Institutional Ethics Committee at Shanghai Chest Hospital. We identified and reviewed the clinical data of patients who were diagnosed with NSCLC at the Shanghai Chest Hospital between January 2011 and December 2013. The study followed the tenets of the Declaration of Helsinki for research involving human subjects. The inclusion criteria were as follows: (1) patients with stage IV NSCLC (NSCLC staging was performed according to the 7^th^ edition of the TNM classification); and (2) patients with the L858R mutation. Patients without treatment and survival details were excluded from this analysis. The baseline clinical characteristics included age at diagnosis, tumor histology, smoking history, and sex.

### Clinical Assessments

EGFR TKIs included icotinib, gefitinib, and erlotinib. Patients were given 150 mg erlotinib daily or 250 mg gefitinib daily, whereas patients who were treated with icotinib received 125 mg three times daily. Clinical follow-up exams were performed every 4–8 weeks. The duration of the PFS was calculated from the date of initiation of EGFR TKI therapy to the date of disease progression or the last follow-up visit. OS was measured from the date of diagnosis until the date of death or the last follow-up visit.

### Test Method For EGFR Mutations

DNA was extracted from five serial slices of a 5-μm paraffin section using the DNA FFPE Tissue Kit (Qiagen, Hilden, Germany). The highly sensitive method termed Amplification Refractory Mutation System (ARMS) was used to detect mutations in the EGFR gene according to the manufacturer’s protocol of the DxS EGFR mutation test kit (DxS)^30^.

### Statistical Methods

Demographic and clinical data are summarized as medians with ranges for continuous variables, and categorical variables are expressed as the means of absolute and percentage numbers. PFS and OS were summarized as median values and two-sided 95% confidence interval (CI), and were analyzed with the Kaplan–Meier method. Any differences between groups were identified with the log-rank test. Statistical significance was defined as P < 0.05. SPSS software, version 22 (SPSS Inc., Chicago, IL, USA) was used for all statistical analyses.

## Additional Information

**How to cite this article**: Xu, J. *et al*. EGFR tyrosine kinase inhibitors versus chemotherapy as first-line therapy for non-small cell lung cancer patients with the L858R point mutation. *Sci. Rep.* **6**, 36371; doi: 10.1038/srep36371 (2016).

**Publisher’s note:** Springer Nature remains neutral with regard to jurisdictional claims in published maps and institutional affiliations.

## Figures and Tables

**Table 1:** Demographic data of all patients.

**Characteristic**	**EGFR TKIs (n = 118)**	**Chemotherapy (n = 127)**	**P**
Median age (range)	67 (30–86)	61 (34–81)	
≥65	67 (56.8%)	42 (33.1%)	<0.001
<65	51 (43.2%)	85 (66.9%)	
Gender			
Male	51 (43.2%)	67 (52.8%)	0.136
Female	67 (56.8%)	60 (47.2%)	
Smoking status			
Smoker	22 (18.6%)	36 (28.3%)	0.074
Never-smoker	96 (81.4%)	91 (71.7%)	
Histology			
Adeno	109 (92.4%)	105 (82.7%)	0.023
Others	9 (7.6%)	22 (17.3%)	
Types of EGFR TKI			
Erlotinib	31 (26.3%)		
Gefitinib	63 (53.4%)		
Icotinib	24 (20.3%)		
Subsequent EGFR TKIs therapy			
Yes		115 (90.6%)	
No		12 (9.4%)	

Abbreviation: EGFR, epidermal growth factor receptor; TKIs, tyrosine kinase inhibitors.
