# Corrigendum: EGFR tyrosine kinase inhibitors versus chemotherapy as first-line therapy for non-small cell lung cancer patients with the L858R point mutation

**DOI:** 10.1038/srep38270

**Published:** 2016-12-01

**Authors:** Jianlin Xu, Haitang Yang, Bo Jin, Yuqing Lou, Yanwei Zhang, Xueyan Zhang, Hua Zhong, Huiming Wang, Dan Wu, Baohui Han

Scientific Reports 6: Article number: 3637110.1038/srep36371; published online: 11 04 2016; updated: 12 01 2016

In this Article, Haitang Yang is incorrectly listed as being affiliated with the ‘Department of Pathology, Shanghai Chest Hospital, Shanghai Jiaotong University, Shanghai 230032, China’. The correct affiliation is listed below:

Department of Thoracic Surgery, Shanghai Chest Hospital, Shanghai Jiaotong University, Shanghai 230032, China.

